# Evaluating health worker performance in Benin using the simulated client method with real children

**DOI:** 10.1186/1748-5908-7-95

**Published:** 2012-10-08

**Authors:** Alexander K Rowe, Faustin Onikpo, Marcel Lama, Michael S Deming

**Affiliations:** 1 Malaria Branch, Division of Parasitic Diseases and Malaria, Center for Global Health, Centers for Disease Control and Prevention, Mailstop A06, 1600 Clifton Road NE, Atlanta, GA, 30333, USA; 2 Direction Départementale de la Santé Publique de l′Ouémé et Plateau, Ministry of Public Health, Porto Novo, B.P. 139, Benin; 3 Africare-Benin, Porto Novo, Benin; 4 Parasitic Diseases Branch, Division of Parasitic Diseases and Malaria, Center for Global Health, Centers for Disease Control and Prevention, Mailstop A06, 1600 Clifton Road NE, Atlanta, GA, 30333, USA

**Keywords:** Benin, Child health, Developing country, Methods, Health services research, Integrated management of childhood illness, Simulated clients

## Abstract

**Background:**

The simulated client (SC) method for evaluating health worker performance utilizes surveyors who pose as patients to make surreptitious observations during consultations. Compared to conspicuous observation (CO) by surveyors, which is commonly done in developing countries, SC data better reflect usual health worker practices. This information is important because CO can cause performance to be better than usual. Despite this advantage of SCs, the method’s full potential has not been realized for evaluating performance for pediatric illnesses because real children have not been utilized as SCs. Previous SC studies used scenarios of ill children that were not actually brought to health workers. During a trial that evaluated a quality improvement intervention in Benin (the Integrated Management of Childhood Illness [IMCI] strategy), we conducted an SC survey with adult caretakers as surveyors and real children to evaluate the feasibility of this approach and used the results to assess the validity of CO.

**Methods:**

We conducted an SC survey and a CO survey (one right after the other) of health workers in the same 55 health facilities. A detailed description of the SC survey process was produced. Results of the two surveys were compared for 27 performance indicators using logistic regression modeling.

**Results:**

SC and CO surveyors observed 54 and 185 consultations, respectively. No serious problems occurred during the SC survey. Performance levels measured by CO were moderately higher than those measured by SCs (median CO – SC difference = 16.4 percentage-points). Survey differences were sometimes much greater for IMCI-trained health workers (median difference = 29.7 percentage-points) than for workers without IMCI training (median difference = 3.1 percentage-points).

**Conclusion:**

SC surveys can be done safely with real children if appropriate precautions are taken. CO can introduce moderately large positive biases, and these biases might be greater for health workers exposed to quality improvement interventions.

**Trial number:**

http://clinicaltrials.gov Identifier NCT00510679

## Introduction

The simulated client (SC) method for evaluating health worker performance utilizes surveyors who pose as patients to make surreptitious observations of health worker practices during consultations. The method has been used for decades in numerous settings [1,2]. Compared to conspicuous observation (CO) by a surveyor in the consultation room, which is commonly done in developing countries, SCs have two key advantages. First, because health workers are unaware of being observed, SC data better reflect usual health worker practices. This information is important because CO can alter health worker practices—often causing performance to be better than usual (*i.e.*, the Hawthorne effect) [3-7]. Second, by using a standardized case history, SCs remove the variation of presenting clinical signs and symptoms that patients naturally have. Thus, SCs could be considered a ‘gold standard’ method [8].

When evaluating performance for pediatric illnesses, a potential drawback of SCs is that clinical guidelines usually require health workers to examine the patient. However, to the best of our knowledge, there are no published accounts of using real children as SCs. Previous SC studies involved surveyors posing as parents who described an ill child who was not actually brought to a health worker [1]. There are, however, potential complications with using real children. If ill children are used to better simulate illnesses, it might be difficult to find children with the appropriate illness and obtain informed consent from the parents; and it might be unethical to delay treatment while planning the SC visit. Also, if healthy children are used, it would be unethical to give them medicines. This issue is relevant because some guidelines (*e.g.*, in the World Health Organization’s Integrated Management of Childhood Illness [IMCI] strategy [9]) require health workers to administer the first dose of a medicine during the consultation.

In the context of a study that evaluated IMCI in Benin [10], we were concerned that the primary data collection method (CO of health workers) would overestimate performance levels. To assess the validity of CO, we wanted to use SCs. The objectives of this study were to evaluate the strengths and limitations of the method and use the results to assess the validity of CO. Regarding SC terminology, while recognizing that both the child and surveyor (the child’s parent) can be considered as SCs, in this report, we refer to the surveyor/parent as the SC and the child as simply the SC’s child.

## Methods

### Study design and setting

The study compared results of two cross-sectional health facility surveys that were conducted in 2002, one after the other, in the same 55 facilities. The first survey used SCs, from 28 August to 4 September; and the second survey used CO, from 16 September to 30 October.

The setting was health facilities in five rural communes (*i.e.*, districts) and one city (Porto Novo) in southeastern Benin. The study was nested in a larger trial that evaluated an intervention package (strengthened supervision, job aids, and non-financial incentives) to improve health worker adherence to IMCI guidelines [10]. At the time of the SC and CO surveys, about one-half of health workers in the study area had received training on IMCI guidelines.

### Inclusion criteria and sampling

For both the SC and CO surveys, inclusion criteria for health facilities were public or licensed private facilities providing outpatient services to children at a level that was appropriate for IMCI. For both surveys, all 55 eligible facilities were included, each facility was visited once, and survey dates were weekdays selected with systematic sampling. With the SC survey, facilities were visited by an SC, her child, a co-surveyor, and a driver (Table 1). With the CO survey, facilities were visited by a team (an observer, interviewer, re-examiner, supervisor, and driver) for one full day during regular working hours to enroll all eligible patients. Inclusion criteria for patients in the CO survey were children 1 week to 59 months old seen in the outpatient setting for any illness.

**Table 1:** Steps in the simulated client (SC) method in a health facility (HF) survey in Benin

1.	The SC and the co-surveyor drove to within 10-minutes walking distance from the HF.
2.	The SC walked to the HF and observed the consultation for her child. The SC did not choose the health worker (HW); she went to whichever consultation room she was told to by the HF staff and seen by the HW there.
3.	During the consultation, the following steps were taken.
	**a.** At the beginning of consultation, the SC gave the child’s health booklet to the HW.
	**b.** The SC spontaneously offered a chief complaint that the child had fever for 1 day.
	**c.** If the HW asked, the SC said the child had diarrhea for 1 day and vomited once. All other signs and symptoms denied.
	**d.** When the time came to purchase medicines, if the HW prescribed an injection, the SC said she had little money and could not buy injections and she asked the HW to write which injections the child needed. All non-injectable medicines were purchased.
	**e.** If the HW tried to give the first dose of a medicine to the child, the SC said she could not give medicines to the child until she showed them to her husband.
	**f.** If the HW ordered a blood test, the SC said she would not agree to have the child’s blood taken because she had to ask her husband first. (During training, SCs were repeatedly instructed to never allow her child to be given a medicine or a blood test.)
4.	The SC walked back to the survey vehicle and began completing the questionnaire.
5.	The co-surveyor walked or drove to the HF and told the HW that he was there to collect information on the children seen that day. This story gave the co-surveyor an excuse to examine HF records. The HW’s identity was confirmed when the name of the SC child was found.
6.	The co-surveyor returned to the survey vehicle and recorded on the questionnaire the HW’s name and whether the register was completely filled-in (correctness not assessed).

### Simulated client survey

All five SCs were women, aged 24 to 43 years (median = 32 years). Four SCs reported past survey experience, and two reported some acting experience. All were fluent in French and two local languages. Six healthy children participated: two boys and four girls, aged 6 to 59 months (median = 39 months). Six children were utilized because one child was briefly ill and temporarily replaced.

The SC scenario was that the child had fever, diarrhea, and one episode of vomiting with no signs of severity or other illnesses. According to IMCI guidelines [11], these symptoms are classified as uncomplicated malaria and diarrhea without dehydration. SCs offered fever spontaneously as a chief complaint, and SCs only mentioned diarrhea and vomiting if the health worker asked. Thus, the scenario included a relatively simple illness and tested the health worker’s competence in asking for key symptoms that were not spontaneously offered, which is recommended by IMCI guidelines.

Three months before the survey, study investigators (AKR, FO, and ML) trained the SCs for three days on observing health worker practices and recording results on a standard three-page questionnaire. The training, which we used to both prepare surveyors and test the methodology, involved: reading the protocol (Table 1) and questionnaire; discussions about what to do if discovered; observing real consultations; practicing the protocol in a classroom setting with role plays, first without children and then with children; refining the protocol and questionnaire after the practice with feedback from SCs; practicing the protocol once in a clinic; and concordance testing. For concordance testing, SCs practiced the methodology, and their recorded responses were compared to ‘gold standard’ responses created by study investigators; the concordance score equaled the percentage of gold standard responses that the SC correctly recorded. After each test, SCs were given constructive feedback. Only two SCs achieved the minimum score of 90%.

Just before the survey, SCs received two days of refresher training. All SCs achieved the minimum performance level when concordance testing was repeated. Final test scores ranged from 96.4% to 100%.

To make the SCs convincing, SCs dressed and spoke like the real patients observed during the training. Also, we provided SCs with a specially prepared child’s health booklet with documentation that the child was up-to-date on vaccinations and a standard set of notes for one fictitious consultation (diarrhea and rash) to make the booklet look used. Different booklets were used for each health facility visit to prevent health worker notes from influencing health worker practices during subsequent SC visits.

Regarding ethical issues, we designed the methodology to protect the health workers that were observed, the SCs, and their children. Health workers were consented in advance. As part of the larger IMCI trial, written informed consent was obtained from all health workers observed during a CO survey in 2001 in the study area. Health workers were informed that they might be visited by SCs. We did not say when SC visits would occur.

To protect SCs, we did the following. First, several discussions were held in a group setting and individually to reassure SCs that they could withdraw at any time. Second, in case an SC was discovered, they carried an official letter from the Ministry of Health authorizing the survey. Third, each SC was accompanied by a co-surveyor who waited near the surveyed facility, out of sight, who could be called upon for help if the child became sick or if there was a conflict with the observed health worker (Table 1). Co-surveyors were clinicians (four physicians and one senior nurse) who were either Ministry of Health or project staff. Fourth, if oral medicines were prescribed, SCs were instructed to buy them, but to refuse to give medicines to the child during the consultation—claiming that she needed her husband’s permission and that she would give them at home. If injections were prescribed, SCs were instructed to not buy them (claiming a lack of money) and to ask the health worker to write the details of the injections in the child’s health booklet so she could buy them later. During training, SCs were repeatedly instructed to never allow their children to be given a medicine or blood test. Finally, SC training included role plays so SCs could practice how to react if they were discovered by a health worker.

Data collection involved visits to two to three facilities per day. Specific steps are presented in Table 1.

### Conspicuous observation survey

The CO methodology is described in detail elsewhere [12]. Briefly, after obtaining consent from health workers and child caretakers (usually the mother), data were collected with five methods: silent observation of consultations with data recorded on a standard checklist (after each consultation, the surveyor asked the health worker for the child’s diagnoses); interviews with caretakers as they left the facility to ascertain prescribed medications and caretakers’ understanding of treatment instructions; re-examination of the child by an expert surveyor clinician, out of the health worker’s view, to obtain a ‘gold standard’ determination of the child’s IMCI illness classifications; health facility assessments to evaluate supplies and equipment; and health worker interviews to obtain information on demographics, training, supervision, and knowledge. After re-examination, inadequately treated children were given appropriate medications without charge. Surveyors were trained until the agreement of practice results of surveyors and study investigators was greater than 90%.

### Analysis

Data were double-entered and verified with EpiInfo version 6 (Centers for Disease Control and Prevention, Atlanta, Georgia). As the standard SC case history was designed to evaluate performance for initial consultations of children 2 to 59 months old, the analysis was restricted to this group. IMCI treatment guidelines vary slightly according to the patient’s age (antimalarial dosages are greater for older children), and the analysis accounted for the age of the child. Analyses were performed with SAS version 9.2 (SAS Institute Inc., Cary, North Carolina). For hypothesis testing, alpha equaled 0.05.

To quantify the bias introduced by CO, with SCs as a gold standard, we calculated absolute differences in the values of child-level performance indicators from the two surveys. To determine if the CO bias was different for health workers with and without IMCI training, CO – SC differences were stratified by the IMCI training status of the worker who performed the consultation. IMCI training status was determined by checking the health worker’s name against a list of IMCI-trained workers.

To evaluate CO bias statistically, the SURVEYLOGISTIC procedure was used to perform logistic regression modeling on all dichotomous indicators. This procedure uses the Taylor expansion method [13] to address clustering, with each facility being a cluster. First, a univariate model was created for each indicator that included a variable for survey method (SC versus CO). The model was run to test for significant differences for consultations done by all health workers, only IMCI-trained workers, and only non-IMCI-trained workers. Second, for each indicator that applied to both IMCI-trained and non-IMCI-trained workers, a model was created with variables for survey method, IMCI training status of the health worker, and a survey method x IMCI training interaction term. The significance test of the interaction term was used to determine whether the CO bias was different for IMCI-trained versus non-IMCI-trained workers. Differences for the one continuous indicator (consultation duration) were analyzed with the Wilcoxon rank sum test, which compares medians, because the distribution of indicator values was highly skewed. Additionally, five sensitivity analyses were performed (Table 2).

**Table 2:** Sensitivity analyses of the simulated client (SC) and conspicuous observation (CO) surveys in Benin

1	An analysis was performed of results for the subset of health workers included in both the SC and CO surveys, to account for differences among workers in the two surveys.
2	SC data were weighted from a given health facility with caseload data from the CO survey at the same facility, to adjust for different sampling schemes (the SC survey included one observation per facility, and the CO survey included multiple observations per facility).
3	Results from both surveys were adjusted for IMCI training status of the health worker who performed the consultation, to adjust for different proportions of children seen by IMCI-trained workers in the two surveys.
4	An analysis was performed that weighted SC data with CO survey caseloads and adjusted for IMCI training status.
5	An analysis was performed that excluded the diarrhea indicators, because diarrhea was ‘hidden’ in the SC case history (*i.e.*, SCs never spontaneously complained of diarrhea, and requiring health workers to ask made SC cases more difficult than diarrhea cases in the CO survey) and if health workers failed to identify the diarrhea, they scored poorly on seven of the 25 performance indicators (*i.e.*, performance for diarrhea had a moderately strong influence on overall estimates of CO bias).

### Ethical approval

The study protocol was approved by the Ethics Committee of the Benin Ministry of Public Health and CDC’s Human Subjects Review Board.

## Results

### Enrollment

Of the 55 eligible health facilities, 47 (85.5%) were public, and 8 (14.5%) were private. In the SC survey, 54 facility visits were successfully made, each with a different health worker. In the one remaining facility, the sole worker on duty (a nursing aide) refused to perform the consultation. The SC was asked to wait for the regular nurse, but after several hours, the nurse did not arrive.

In the CO survey, 55 health facility visits were completed. Initial consultations were observed for 185 children 2 to 59 months old, who were seen by 59 different health workers in 46 facilities. In the nine other facilities, although survey teams were in place for the full day, no initial consultations for children 2 to 59 months old occurred.

Altogether, the performance of 89 different health workers was assessed: 24 were in both the SC and CO surveys, 30 were only in the SC survey, and 35 were only in the CO survey. As per the SC scenario, all SC children had fever and diarrhea. In the CO survey, according to the gold standard surveyor’s re-examination, 142 children had a febrile illness and 27 had diarrhea. Regarding spontaneously offered chief complaints, among the 142 children with a febrile illness, 69.7% of caretakers complained of fever. Among the 27 children with diarrhea, 55.6% of caretakers complained of diarrhea. In contrast, all SCs complained of fever, and none complained of diarrhea. The proportions of children seen by IMCI-trained health workers in the SC and CO surveys were similar: 42.6% (23/54) and 47.0% (87/185), respectively.

### Process of the simulated client survey

No serious problems occurred during the SC survey. Minor problems involving SCs and their children included: the health facility visits tired out the children somewhat; one child vomited several times in the car ride to a facility, probably due to motion-sickness; one child had a very minor injury when a tongue depressor broke in his mouth during an examination; and one child developed a non-severe febrile illness that resolved three days after treatment with chloroquine (the recommended antimalarial at the time of the study). Observed health workers indicated that our surveyors were SCs in two (3.7%) of the 54 visits, although there were no confrontations. One problem involving the observed health workers was that 31 workers visited by SCs had not been previously consented. When we discovered this issue immediately after the survey, we reported it to our human subjects board, and project staff visited the workers to request written, informed consent. All 31 health workers agreed to participate, and their results were included.

### Validity assessment of conspicuous observation

**Figure 1:**
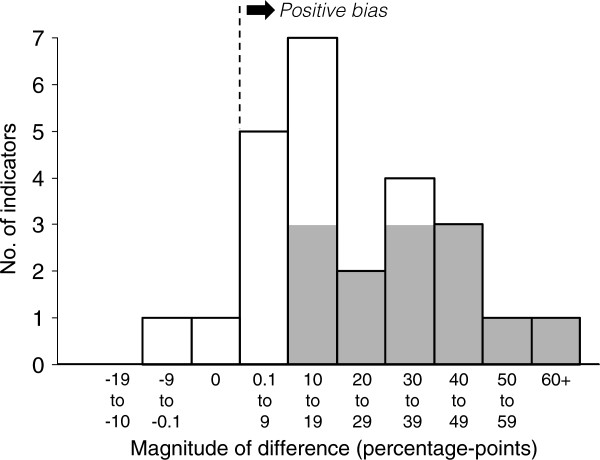
**Validity of the conspicuous observation survey method for 25 performance indicators in Benin: simple comparison of the conspicuous observation and simulated client surveys (*i.e.*, conspicuous observation survey result minus simulated client survey result; see Table**3**, column 7).** NB. Shading of the vertical bars indicates statistically significant differences.

Table 3 presents indicator values, stratified by survey method and health worker IMCI training status. The first assessment of CO bias, compared to an SC gold standard, was the absolute difference in indicator values between the two surveys (column seven). Nearly all CO – SC differences were positive, and differences for 13 of the 25 indicators were statistically significant. The distribution of differences was wide, ranging from −1.7 to 61.1 percentage-points (%-points) (Figure 1); and the median difference was moderately large (16.4%-points).

**Table 3:** Comparison of simulated client and conspicuous observation methods for health facility surveys in Benin

**Indicator (all indicators measured as percentages, unless otherwise stated)**	**Simulated client survey**			**Conspicuous observation survey**			**Difference between methods (conspicuous observation – simulated client)**			**Difference for HWs with IMCI – difference for HWs without IMCI (column 8 – column 9) [Column 10]**
	**All HWs [Column 1]**	**HWs with IMCI [Column 2]**	**HW without IMCI [Column 3]**	**All HWs [Column 4]**	**HWs with IMCI [Column 5]**	**HW without IMCI [Column 6]**	**All HWs (column 4 – column 1) [Column 7]**	**HWs with IMCI (column 5 – column 2) [Column 8]**	**HW without IMCI (column 6 – column 3) [Column 9]**	
Median consultation duration, in minutes	16	19	15	21	29	15	5 (relative difference = 31.3%)	10 (relative difference = 52.6%)**	0 (relative difference = 0%)	10 (difference of relative differences = 52.6%)**
HW offered greeting	55.6	56.5	54.8	92.4	96.6	88.8	36.8***	40.1***	34.0**	6.1
Consultation performed by IMCI-trained HW	42.6	NA	NA	47.0	NA	NA	4.4	NA	NA	NA
For children seen by IMCI-trained HW…										
The IMCI chart booklet was used	NA	43.5	NA	NA	92.0	NA	NA	48.5**	NA	NA
An IMCI patient recording form was used^a^	NA	56.5	NA	NA	85.1	NA	NA	28.6*	NA	NA
HW determined^b^ if child…										
Was unable to drink or breastfeed	22.2	34.8	12.9	37.8	69.0	10.2	15.6	34.2*	–2.7	36.9
Was vomiting everything	46.3	65.2	32.3	48.6	71.3	28.6	2.3	6.1	–3.7	9.8
Had convulsions	20.4	39.1	6.5	38.9	78.2	4.1	18.5*	39.1***	–2.4	41.5*
Had cough or difficult breathing	64.8	82.6	51.6	89.7	95.4	84.7	24.9***	12.8	33.1***	–20.3
Had diarrhea	33.3	56.5	16.1	67.0	93.1	43.9	33.7***	36.6**	27.8*	8.8
Had ear problem	27.8	60.9	3.2	40.5	72.4	12.2	12.7	11.5	9.0	2.5
Child was weighed	90.7	95.7	87.1	96.2	96.6	95.9	5.5	0.9	8.8	–7.9
Temperature taken	100.0	100.0	100.0	100.0	100.0	100.0	0	0	0	0
HW checked for palmar pallor	44.4	82.6	16.1	50.8	90.8	15.3	6.4	8.2	–0.8	9.0
For children with fever…										
Child checked for neck stiffness	11.1	21.7	3.2	32.0	57.6	11.1	20.9*	35.9*	7.9	28.0
Child checked for measles history in past 3 months	14.8	34.8	0	27.9	62.2	0	13.1	27.4	0	27.4
The illness is correctly classified^c^	62.3	77.3	51.6	78.2	84.6	72.7	15.9*	7.3	21.1	–13.8
The illness is correctly treated^c^	47.2	90.9	16.1	45.5	70.7	22.2	–1.7	–20.2	6.1	–26.3
For children with diarrhea…										
HW asks for duration	31.5	52.2	16.1	74.1	84.2	50.0	42.6**	32.0	33.9*	–1.9
HW asks if bloody stools	3.7	8.7	0	55.6	63.2	37.5	51.9***	54.5***	37.5	17.0
HW offers drink to assess thirst	1.9	4.4	0	48.1	68.4	0	46.2***	64.0***	0	64.0***
HW does skin pinch	5.6	8.7	3.2	44.4	63.2	0	38.8***	54.5**	–3.2	57.7**
The illness is correctly classified^c^	3.7	8.7	0	44.4	63.2	0	40.7***	54.5**	0	54.5**
The diarrhea is correctly treated^c^	9.3	8.7	9.7	70.4	79.0	50.0	61.1***	70.3***	40.3*	30.0
HW tells at least one diagnosis to the caretaker	20.4	26.1	16.1	25.7	41.8	12.4	5.3	15.7	–3.7	19.4
HW advises caretaker to bring the child back immediately if the child…										
Is unable to drink	14.8	34.8	0	25.0	55.2	0	10.2	20.4	0	20.4
Becomes sicker	16.7	39.1	0	33.1	65.7	6.2	16.4*	26.6	6.2	20.4

HW = health worker; IMCI = Integrated Management of Childhood Illness; NA = not applicable.

^a^ An IMCI patient recording form could either be the standard form developed by the World Health Organization or the IMCI patient register, which was a job aid specific to Benin that was being evaluated in a study (Rowe et al., 2009a).

^b^ ‘Determined’ means the health worker was exposed to the information, either because the health worker specifically asked, the caretaker spontaneously offered the information (*e.g.*, as part of the chief complaint), or the child obviously had the sign (*e.g.*, a child actively coughing).

^c^ ‘Correct’ means the health worker’s diagnosis or treatment exactly matched IMCI recommendations, according to the gold standard of the simulated client history (for the simulated client survey) or surveyor re-examiner (for the conspicuous observation survey).

* P-value <0.05.

** P-value <0.01.

*** P-value <0.001.

**Figure 2:**
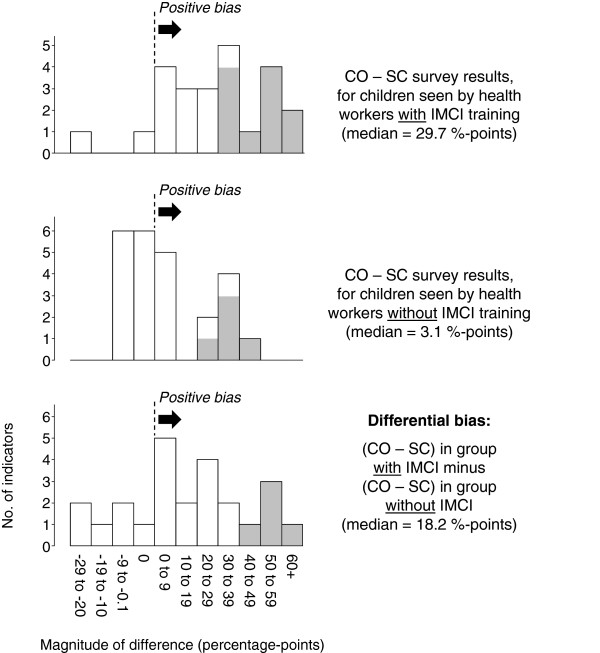
**Validity of the conspicuous observation survey method for 24 performance indicators in Benin: differential bias according to the IMCI training status of the health worker who performed the consultation (see Table**3**, columns 8–10).** CO = Conspicuous observation; IMCI = Integrated Management of Childhood Illness; SC = simulated client. NB. Shading of the vertical bars indicates statistically significant differences.

The second assessment of CO bias stratified CO – SC differences by the IMCI training status of the worker who performed the consultation. In the IMCI group, differences were generally large (median = 29.7%-points, range: –20.2 to 70.3%-points) (column eight of Table 3, and top histogram in Figure 2). In contrast, differences in the non-IMCI group were generally small (median = 3.1%-points, range: –3.7 to 40.3%-points) (column nine of Table 3, and middle histogram in Figure 2). Thus, the differential CO bias (*i.e.*, difference in the IMCI group minus difference in the non-IMCI group) was moderately large (median = 18.2%-points, range: –26.3 to 64.0%-points) (column 10 of Table 3, and bottom histogram in Figure 2).

For the two performance indicators that could only be evaluated for IMCI-trained health workers (use of the IMCI booklet of clinical algorithms and patient recording form; Table 3, rows four and five), we found significant evidence of a large positive CO bias: 48.5 and 28.6%-point differences, respectively.

### Sensitivity analyses

The analysis of the 24 health workers in both surveys (Table 2, sensitivity analysis one) included 95 consultations. With the simple CO – SC estimate of CO bias, we found the same trend of positive bias as the primary analysis, but with a smaller magnitude (median difference = 7.8%-points). For estimates of the differential bias by IMCI-training status, median biases from the primary and sensitivity analyses were very similar: 18.2 and 19.0%-points, respectively.

When SC data were weighted by CO caseload, estimates of CO bias were somewhat greater than those from the primary analysis: the median difference was 21.2%-points without adjusting for IMCI training (sensitivity analysis two) and 22.9%-points with adjusting for IMCI training (sensitivity analysis four). When only adjusted for IMCI training (sensitivity analysis three), results were very similar to those from the primary analysis (median difference = 17.2%-points).

When diarrhea indicators were excluded (sensitivity analysis five), estimates of the median CO bias were lower than those in the primary analysis: 12.9%-points for all health workers, 15.7%-points among IMCI-trained workers, and 0%-points among workers without IMCI. The median of the IMCI versus non-IMCI differential bias was 9.8%-points.

### Survey costs

Field costs per health facility visit were quantified in terms of person-days for staff (*i.e.*, surveyors and drivers) and ‘vehicle-days.’ Calculations included surveyor training and the actual survey in 55 facilities. The SC method required 1.77 person-days for staff and 0.54 vehicle-days per facility visit, and the CO method required 7.33 person-days for staff and 1.18 vehicle-days per facility visit (*i.e.*, CO–SC difference = 5.56 person-days for staff and 0.64 vehicle-days per facility visit). Given average daily staff and vehicle costs during the survey ($27.27 and $40.60, respectively, in 2002 US$), the average field costs per facility visit of the CO survey ($247.80) were 3.5 times greater than in the SC survey ($70.19). However, the number of included consultations per facility visit was 3.4 times (*i.e.*, 185/54) greater in the CO survey. Thus, the cost per consultation in the CO and SC surveys was similar: $73.67 and $70.19, respectively.

## Discussion

In the context of an IMCI trial in Benin, we tested the SC method with real children to evaluate health worker performance and used the results to assess the validity of CO. The first main finding was that the SC method generally worked well. Of the few problems encountered, none was serious. SCs were discovered in only 3.7% of visits, which is comparable to other studies (about 5% to 10%) [14].

The second main finding was the poor performance of many health workers for many basic recommended clinical tasks. However, the particularly striking finding from the SC survey was that most health workers (even IMCI-trained workers) failed to ask about diarrhea when it was not spontaneously offered as a complaint; and that failure led to a cascade of errors, ultimately resulting in incorrect treatment for virtually all diarrhea cases. Although this chain of events might not be surprising, it is a stark reminder of how skipping even simple tasks can lead to potentially life-threatening errors. Clearly, IMCI training, supervision, and other health worker support activities should focus on systematically asking for main symptoms. Perhaps more generally, those who design and implement clinical guidelines should identify in advance ‘gateway’ tasks at the beginning of an algorithm, which if skipped or done incorrectly could lead to a clinically serious error cascade; and adherence to these tasks should be emphasized.

The third main finding was that, as expected, CO results were positively biased for most performance indicators. Although CO – SC differences varied by indicator, the median difference was moderately large (16.4%-points). Sensitivity analyses that adjusted for differences between the CO and SC surveys produced median differences that varied somewhat, from 7.8 to 22.9%-points; but all results supported the same conclusion. These results are comparable to the peak effect of CO (13%-points) from a Tanzanian study that compared CO to patient interviews [4]. And our median difference of 16%-points was identical to results from a Kenyan study that compared CO of community health workers in a hospital setting to a review of patient registers that the workers routinely filled out while treating patients in their villages [7].

The fourth main finding was that the CO bias seemed much larger for IMCI-trained health workers than for non-IMCI-trained workers (median difference between groups = 18.2%-points). However, we are cautious about these results because our sample size was relatively small, and significant differences were found for only five indicators. Also, for the two treatment indicators, which are probably most closely linked to patient outcomes, the estimates of differential bias were contradictory: a positive bias for diarrhea (30.0%-points) and a negative bias for febrile illness (−26.3%-points). Still, to the best of our knowledge, this study is the first to evaluate differential CO bias as a function of exposure to a quality improvement intervention (IMCI, in our case). Perhaps, compared to non-IMCI-trained workers, the IMCI-trained workers were more aware of the standard they were expected to follow; and this awareness translated into greater pressure to conform to the standard during CO. A related explanation is that several CO surveyors were IMCI trainers; and some workers might have exerted greater effort to follow IMCI guidelines if they had been observed by someone who had trained them. Another possibility is that CO caused health workers to use IMCI job aids more often (Table 3, rows four and five), which then caused improved performance. If this differential bias is confirmed, then trials to improve health worker performance that use CO might have positively biased results.

Although we used SCs as a gold standard, the method is not perfect. Advantages and disadvantages of SCs have been mentioned and are discussed in detail elsewhere (see reference 1 and the on-line Additional file 1: Appendix). In particular, the disadvantages highlight a deeper issue about assessing health worker performance. Although many methods exist (*e.g.*, CO, SCs, health worker knowledge tests and vignettes, chart review, patient re-examination, exit interviews, and health worker self-assessment), there is relatively little evidence-based guidance on: which method or combination of methods is best for which purpose (*i.e.*, routine program monitoring versus research, for a variety of health topics in a variety of settings); and what are the best practices for each method. Moreover, relatively little methodological research has been done in developing countries to answer these questions.

One other method deserving special attention is reviewing videotaped health worker–patient encounters. This method, primarily applied in high-income countries, has been used in studies of trauma resuscitations [15-17], anesthesia [18], cardiac arrest [19], patient examinations [20], health worker–patient communication [21-24], and nursing workload [25]. Although for technical and ethical reasons it would be difficult to videotape without health workers’ knowledge, when done long enough for health workers to become accustomed to it, the results might reflect usual practices [26]. Alternatively, if used continuously (*e.g.*, in trauma centers, for education and quality improvement [27]), the results always portray usual practices. In developing countries, videotaping could be used. However, in some settings, such as small rural clinics, maintaining equipment and obtaining patient consent could be major challenges. Also, while the influence of videotaping might not be great in high-income settings, some researchers working in developing countries have expressed concerns that it could have a large influence [28].

### Other lessons learned

First, it was helpful to add co-surveyors to the SC method to collect data on health worker and facility attributes and to protect SCs and their children. Second, it was useful having a ‘hidden’ symptom (*e.g.*, diarrhea) in the SC case history. It revealed how well health workers managed illnesses when caretakers did not spontaneously offer symptoms. Also, by having one ‘obvious’ and one ‘hidden’ illness, the SC case history would have two illness classifications per child—which is typical for IMCI [12,29]. However, future surveys should use a hidden symptom that is highly prevalent in the underlying population. In our setting, because few children were brought to health facilities with diarrhea, our assessment of CO bias for the hidden illness was limited by small sample size. A better choice would have been cough, which was much more common. On the same topic, a third lesson was that if a proposed hidden symptom in the SC case history is often spontaneously offered by real patients, then it might be wise to have two SC case histories: one in which the SC does not spontaneously give the complaint, and one in which the SC does. For example, because about one-half of caretakers of children with diarrhea spontaneously gave the complaint (from our CO survey), we could have created two SC case histories, with and without the complaint; and with two SC visits per facility (by two different SCs, each with a different case history), the SC data would have more closely matched the reality that health workers actually faced.

### Study limitations

Our study had five main limitations. First, the sample size was relatively small, especially for diarrhea indicators. Thus, in the analysis of differential CO bias by IMCI training status, most differences were not statistically significant. However, if there was truly no differential CO bias, then the distribution of the 24 indicator-specific differences would have been centered more closely around zero. The second main limitation (also related to the differential CO bias by IMCI training status) was that some health workers might have been observed by clinicians who had trained them in IMCI, which might have actually caused some bias. Third, only one-quarter of health workers were assessed by both methods. Fourth, as the minimum level of surveyor performance was 90% and because observing consultations is often complex, it is likely that some data were erroneous. Fifth, the SC standard case history’s hidden illness (diarrhea) probably made it more difficult than diarrhea cases in the CO survey, and thus overestimated CO bias.

## Conclusions

SC surveys can safely be done with real children, if appropriate precautions are taken. As expected, CO can introduce moderately large positive biases; however, these biases might be greater for health workers exposed to quality improvement interventions. Thus, although confirmation is needed, CO might overestimate intervention effectiveness for some performance outcomes.

## Competing interests

The authors declare that they have no competing interests.

## Authors’ contributions

AKR and MSD conceived of the study. All authors developed the protocol. AKR, FO, and ML coordinated the field work. AKR analyzed the data and helped draft the manuscript. All authors contributed substantially to the writing and editing of the manuscript, and all authors read and approved the final manuscript.

## Supplementary Material

Additional file 1 **Appendix 1.** Advantages and Disadvantages of the Simulated Client Method. Click here for file
