# Supplementary material for: Evaluating health worker performance in Benin using the simulated client method with real children
Source: Implement Sci. 2012 Oct 8;7:95. doi: 10.1186/1748-5908-7-95 (PMC3541123; doi:10.1186/1748-5908-7-95)
Supplement: Additional file 1 — Appendix 1. Advantages and Disadvantages of the Simulated Client Method. [file 1748-5908-7-95-S1.doc]

**On-line appendix to the article:** Evaluating health worker performance in Benin using the simulated client method with real children.

Authors: Alexander K Rowe, Faustin Onikpo, Marcel Lama, and Michael S Deming

**Advantages and Disadvantages of the Simulated Client Method**

*Advantages*

First, health workers (usually) do not know they are being observed, thus there is a lower chance that the Hawthorne effect would bias assessments. Second, simulated client (SC) case histories can be standardized to reduce the influence of case mix on quality assessments. Third, in settings like rural Benin, where occasionally there are no children seen in some facilities, the SC method can increase the sample of facilities in which performance is evaluated. Fourth, the SC method might be less expensive than conspicuous observation (CO) for the same number of facilities, although the sample size of consultations would probably be much less with the SC method.

*Disadvantages*

First, SC children are not real patients, which could introduce bias. For example, healthy SC children might be easier or more difficult to examine than sick children; and as SCs are not actually worried that their child is ill, they might be less demanding than parents of truly ill children. Additionally, as the standard case history means that SCs either always or never spontaneously offer a complaint of a particular symptom, bias could be introduced if spontaneous complaints influence (probably improve) health worker performance. For example, as our SCs always spontaneously complained of fever but never diarrhea, management of SC children would theoretically be easier for fever indicators and more difficult for diarrhea. In our particular study, this issue was probably not important for fever, because a complaint of fever was not associated with diagnosis or treatment quality; but it probably was important for diarrhea, because diarrhea diagnosis and treatment seemed to be better when a complaint of diarrhea was given—although sample sizes were too small to be conclusive (results not shown).

Second, it would be difficult or impossible for SCs to simulate some health conditions (e.g., severe illnesses, illnesses diagnosed with an invasive laboratory test, and illnesses with an expected clinical sign, such as a bulging fontanelle or actual fever). This is an important limitation because some data suggest that the performance of IMCI-trained workers for severe illnesses has been lower than for moderate illnesses [1]. Thus, what is learned in an SC survey about moderate illness might not be generalizable to severe illness.

Third, the method might not work in settings where social or cultural aspects of a client are difficult to simulate (e.g., community health workers who know everyone in their village might be suspicious of SCs from outside the community). Fourth, SCs by themselves cannot collect much information about health worker and facility attributes (e.g., health worker training, recent supervision, and availability of commodities). Our approach of implementing SCs with a co-surveyor who could collect such information might be a useful model for future surveys.

Fifth, the SC method with real children creates the possibility that a child could be harmed during a facility visit. Sixth, in the small proportion of visits in which SCs are discovered, the key benefit of SCs (no Hawthorne effect) is lost, and results could be positively biased. However, if the discovery occurs near the end of the consultation, the bias might be less. Seventh, in malarious areas where confirmation of malaria with a blood test is required, the ethical use of healthy children is questionable. Perhaps SCs could be used to determine if health workers order tests (several recent studies have found that many patients who should be tested are not [2,3]), but then SCs would need to refuse testing to avoid harming their child. Finally, when the SC method includes real children, one should expect more sick days than the SC method without real children—simply because young children get sick more often than adults.

References

1. Centers for Disease Control and Prevention: **Health worker performance after training in Integrated Management of Childhood Illness — Western Province, Kenya, 1996–1997.** *Morb Mort Weekly Report*, 1998, **47**:998–1001.

2. Rowe AK, Ponce de León GF, Mihigo J, Santelli ACFS, Miller NP, Van-Dunem P : **Quality of malaria case management at outpatient health facilities in Angola.** *Malar J* 2009, **8**: 275.

3. Skarbinski J, Ouma P, Causer L, Kariuki S, Barnwell J, Alaii J *et al*: (2009). **Effect of malaria rapid diagnostic tests on the management of uncomplicated malaria with artemether-lumefantrine in Kenya: A cluster randomized trial.** *Am J Trop Med Hyg* 2009, **80**:919–926.
